# Relationship Between Family Socioeconomic Status and Learning Burnout of College Students: The Mediating Role of Subjective Well-Being and the Moderating Role of Resilience

**DOI:** 10.3389/fpsyg.2022.844173

**Published:** 2022-05-27

**Authors:** Wenzhi Wu, Yilin Liu, Lei Yu, Zhichao Guo, Shujun Li, Zeyi Guo, Xiang Cao, Fangjun Tu, Xiaoqin Wu, Xiao Ma, Qing Long, Xinling Zhao, Xiujuan Li, Yatang Chen, Yong Zeng

**Affiliations:** ^1^The Second Affiliated Hospital of Kunming Medical University, Kunming, China; ^2^The Sixth Affiliated Hospital of Kunming Medical University, Yuxi, China; ^3^Department of Psychiatry, Shenzhen Mental Health Center, Shenzhen, China

**Keywords:** family socioeconomic status (FSES), learning burnout, subjective well-being, resilience, college students

## Abstract

**Objective:**

Learning burnout affects the positive development of college students. The present study aimed to investigate the relationship between family socioeconomic status (FSES) and learning burnout, as well as the mediation effect of subjective well-being and the moderation effect of resilience in this relation.

**Methods:**

A total of 550 Chinese college students from Yunnan completed a questionnaire measuring the research variables in this study.

**Results:**

(1) After controlling for participants’ gender and age, FSES negatively, and significantly predicted learning burnout; (2) subjective well-being partially mediated the relationship between FSES and learning burnout; and (3) the direct effect of FSES on learning burnout and the mediation effect of subjective well-being was moderated by resilience. The level of learning burnout of individuals with low resilience increased significantly with the decrease of FSES, and the level of learning burnout of individuals with high resilience decreased significantly with the increase in subjective well-being.

**Conclusion:**

The present findings support the moderated mediation model underlying the relationship between FSES and learning burnout. This also has significant implications for formulating prevention and intervention measures on learning burnout among college students.

**Limitations:**

First of all, this study used the cross-sectional study design, which cannot make a causal inference. In addition, the sample in this study is university students from Kunming, which may affect the popularity of the results.

## Introduction

Learning burnout is a stress response that students are unable to cope with academic stress, and is an emotional, attitude, and behavioral failure that occurs when students are unable to meet their learning needs (Tuominen-Soini and Salmela-Aro, 2014). Learning burnout has a significant impact on learning and life, leading to a range of adverse developmental outcomes, such as low academic performance, truancy, absenteeism, and dropping out of school (Bask and Salmela Aro, 2013). Family socioeconomic status (FSES) is an important factor in learning burnout, and students with high FSES are more enthusiastic and mentally full in the learning process (Randolph et al., 2006). Consequently, students with high FSES tend to have less burnout (Luo et al., 2016; Yun et al., 2016). Along with the Family Investment Model, households with higher socioeconomic status have significant advantages in terms of income, social capital, and human capital, which can be translated into resources for student development, thereby affecting students’ level of development (Conger and Donnellan, 2007), which in turn leads to a more positive attitude toward learning tasks, resulting in lower learning burnout (Randolph et al., 2006). Previous studies have focused on the relationship between low FSES and adolescent problem behavior, while there have been relatively few studies on the internal mechanism and protective factors of FSES affecting college students’ learning burnout, so it is not possible to mitigate the negative effects of disadvantage on college students’ learning burnout from the front end and prevent behavioral problems. The university period is an important for individuals to acquire knowledge, develop and form self-identity. Therefore, it is of great significance to explore the mechanism and influence conditions of FSES on learning burnout in the group of college students to promote their development.

The Family Stress Model shows that parents with high FSES have less mental and financial stress, more harmonious family relationships, and are more able to raise their children positively, giving them higher subjective well-being and life satisfaction so that there are more positive emotions toward study and life, which has an important impact on reducing students’ learning burnout (Conger et al., 2010; Buzzai et al., 2020). Subjective well-being is defined as the individual’s overall perception and judgment of his or her quality of life based on his or her subjective criteria, characterized by subjectivity, stability, and integrity (Diener et al., 1999), which plays an important role in the formation and development of individual career expectations (Chen and Zhou, 2003). Subjective well-being is a multi-layered and multi-dimensional construct consisting mainly of life satisfaction and emotional experiences (positive and negative emotions) (Wu, 2000). Life satisfaction is an overall cognitive judgment of the quality of life, and the balance of positive and negative emotions reflects the individual’s emotional experience of life (Diener and Ryan, 2009). Studies have shown a significant positive correlation between FSES in adolescent families and subjective well-being and adolescent life satisfaction (Ni et al., 2016; Huang et al., 2019). Low FSES is a high-risk factor for poor emotional adaptation among mobile adolescents (Wang and Mesman, 2015; Li et al., 2016a,b; Yanbin et al., 2016; Zhang et al., 2017), and is closely related to different emotional symptoms in adolescents (Bradley and Corwyn, 2002; Chen et al., 2016). Moreover, there was a significant negative correlation between the subjective well-being of college students and the dimensions of dejection, improper behavior strength, and reduced personal accomplishment of learning burnout (Li et al., 2012). Subjective well-being is an individual’s inclusive evaluation of their quality of life, so individuals with high subjective well-being have more positive inner experiences, enough social support and psychological energy to actively cope with academic pressure, thereby relieving depression and maintaining a high level sense of achievement (An and Zhang, 2016). Therefore, the predictive effect of FSES on the level of college students’ learning burnout may be realized by influencing subjective well-being, explicitly, subjective well-being has a mediating role in the influence of FSES on college students’ learning burnout, but there is still no research to confirm this view.

The Resilience Framework Theory believes that the individual’s protective factors can reduce the negative impact of the risk factors in the external situation on the individual’s development through some means such as selective perception, reconstruction, changing the environment, and active coping (Kumpfer, 1999). Resilience refers to the psychological process by which an individual can still gain positive adaptation when he or she is exposed to a major threat or trauma (Cakir et al., 2021). It is considered a good personal quality that can still perform well after a setback and plays an important role in dealing with external stress situations. Resilience has a direct predictive effect on the individual’s ability to learn independently and is the main factor affecting the adaptation of an individual learning career. Improving resilience can reduce the production of learning burnout (Rios-Risquez et al., 2016; An et al., 2019). Thus a high level of mental resilience can alleviate the risk of learning burnout caused by low FSES (Schelvis et al., 2014; Bird and Pincavage, 2016; McKenna et al., 2016); and the risk of the internal psychological elasticity factors of an individual including emotions (emotional expression and subjective well-being, etc.), cognition (learning and imagination, etc.) and behavior (execution ability, operation ability, etc.) through the process of psychological resilience, so that the individual finally obtains the result of adaptation (Kumpfer, 1999), which means learning burnout may be affected by the interaction of resilience with FSES and subjective well-being. Previous studies have examined the psychological resilience process of cognitive and behavioral factors that affected learning burnout (Wu et al., 2016; Fan et al., 2021), while less exploring the interaction of emotional factors such as subjective well-being and resilience on college students’ learning burnout.

**FIGURE 1:**
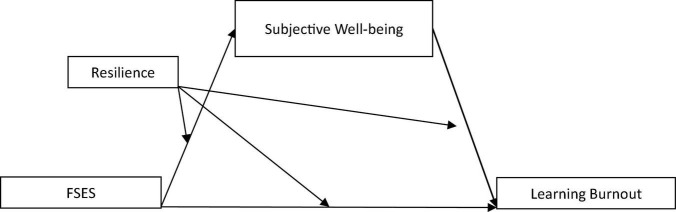
Moderated mediation model regarding perceptions of FSES and learning burnout, mediated by subjective well-being and moderated by resilience.

To sum up, some studies have shown that FSES is an important factor affecting college students’ learning burnout, but the relationship between FSES and college students’ learning burnout and “how and when” FSES affects learning burnout still need to be further explored (see Figure 1). The individual-environmental interaction theory also points out that the individual’s behavioral problems are the result of the interaction of negative environmental factors and individual trait factors (Lerner et al., 2006). Therefore, to further clarify the formation and development mechanism of college students’ learning burnout, it is necessary to examine the mediating and moderating mechanism of environmental factors (FSES), emotional factors (subjective well-being), and cognitive factors (resilience) in college students’ learning burnout from the perspective of multi-factor integration. Based on the above discussion, this study puts forward the following hypotheses:

**H1:** Subjective well-being plays a mediating role between FSES and learning burnout among college students;
**H2:** Resilience plays a moderating role in the mediating model of “FSES—subjective well-being—learning burnout” of college students.

## Materials and Methods

### Participants

In the convenient sampling method, 600 college students were randomly selected for a cross-sectional questionnaire survey at a university in Kunming, Yunnan Province, and a total of 586 paper questionnaires were recovered. There were 550 valid questionnaires obtained after excluding incomplete questionnaires and gender-missing questionnaires with an effective rate of 93.86%. The sample included 200 boys (36.4%) and 350 girls (63.6%). Participants ranged in age from 17 to 26 years of age, with an average age of 19.927 years (SD = 1.559). Other demographic characteristics of the sample were shown in Table 1.

**TABLE 1:** Sociodemographic characteristics of the sample (*n* = 550).

Age (years, *M*/SD)	19.927 (1.559)
**Gender (*n*/%)**	
Boys	200 (36.4)
Girls	350 (63.6)
**Grade (*n*/%)**	
Freshman	146 (26.5)
Sophomore	270 (49.1)
Junior	90 (16.4)
Senior	10 (1.8)
Five-grade	4 (0.7)
Graduate student	28 (5.1)
No information	2 (0.4)
**Academic record (*n*/%)**	
A	14 (2.5)
B	96 (17.5)
C	392 (71.3)
D	32 (5.8)
E	16 (2.9)
**Student cadre (*n*/%)**	
Yes	110 (20.0)
No	432 (78.5)
No information	8 (1.5)
**Only child (*n*/%)**	
Yes	164 (29.8)
No	386 (70.2)
**Family income level (monthly, *n*/%)**	
Up to 2,000 yuan	14 (2.5)
2,000–6,000 yuan	38 (6.9)
6,000–10,000 yuan	112 (20.4)
10,000–14,000 yuan	208 (37.8)
More than 14,000 yuan	178 (32.4)

The questionnaire and methodology for this study were approved by the Human Research Ethics Committee of the Kunming Medical University (Ethics approval number: 2021kmykdx6f65).

### Measures

#### Family Socioeconomic Status

Scholars generally use the three variables of household income, parents’ education level, and occupation to synthesize the FSES index (Bradley and Corwyn, 2002; Xu et al., 2009; Ren and Xin, 2013). The household income surveyed in this study is the monthly household income. Regarding previous studies (Xu et al., 2009), and combined with the actual situation of the local economy, we divide the monthly household income into “below 2,000 yuan,” “2,000–6,000 yuan,” “6,000–10,000 yuan,” and “10,000–14,000 yuan,” and “14,000 yuan or more” five levels and scored 1–5, respectively. Parents’ education level includes “primary school or below,” “junior high school (including those who have not graduated),” “high school or technical secondary school (including those who have not graduated),” “college (including evening universities and TV universities),” “postgraduate (Master or Ph.D.)” five categories and scored 1–5, respectively. Parents’ occupations include “farmers,” “workers,” “doctors,” “teachers or scientific researchers,” “government officials or civil servants,” “lawyers,” “engineers,” “business managers,” “accountants,” and “soldiers,” “individual/private business owners,” “self-employed or laid-off workers,” “freelance workers,” and “others.” For the division of parents’ occupational levels, the present study selected the ten strata proposed by Xia et al. (2017) and scored 1–10 respectively according to occupational level. The higher the score, the higher the occupational social status (Xia et al., 2017). Concerning related studies (Bradley and Corwyn, 2002; Shi and Shen, 2007; Ren and Xin, 2013), we converted the scores of household income, parents’ education level, and parents’ occupations into standard scores and added them together as FSES scores.

#### Subjective Well-Being

The Subjective Well-Being Questionnaire was compiled by Diener including life satisfaction (5 items, e.g., “I am satisfied with my life”) (Diener et al., 1985), positive affect frequency (6 items, e.g., “treat”), and negative affect frequency (8 items, e.g., “treat”) (Watson et al., 1988). All three scales are scored by 7 points. The total score of subjective well-being is calculated by adding positive emotions to life satisfaction minus negative emotions after averaging the three scales separately (Sheldon and Elliot, 1999). Studies have proved that it has good reliability and validity in the context of Chinese culture (Yang et al., 2020; Tian et al., 2022). In this study, the CFA model of life satisfaction scale generated a very good fit, with χ^2^*/df* = 1.428, *p* < 0.001, SRMR = 0.032, RMSEA = 0.055, GFI = 0.980, and TLI = 0.961, the Cronbach’s alpha coefficients of three weight scales were 0.793, 0.825, and 0.899.

#### Learning Burnout

The Learning Burnout Scale (LBS) comprises 20 items including three dimensions: dejection (reflects that college students are unable to deal with the problems and requirements in their studies well, e.g., “I feel exhausted after studying all day”), improper behavior strength (reflects that college students are tired of studying thus behaving characteristics such as skipping class, not attending class, being late, leaving early, not handing in homework, etc., e.g., “I seldom study after class”), reduced personal accomplishment (reflecting college students’ feelings of low achievement in the learning process, e.g., “The mastery of professional knowledge is easy for me”). The questionnaire used a five-point Likert scale ranging from 1 (completely disagree) to 5 (completely agree). Responses across the 20 items were averaged to obtain the score, with higher scores indicating a higher degree of learning burnout (Lian et al., 2005). In this study, the second-order CFA model generated a very good fit, with χ^2^*/df* = 4.146, *p* < 0.001, SRMR = 0.038, RMSEA = 0.058, GFI = 0.944, and TLI = 0.909, and both the absolute and value-added adaptation indexes were in the acceptable range. The Cronbach’s alpha coefficient for the Connor-Davidson Resilience Scale was 0.835.

#### Resilience

The Connor-Davidson Resilience Scale consists of 25 items and measures three aspects of resilience: (1) tenacity (refers to a person’s composure, alertness, perseverance, and sense of control in the face of difficulties and challenges, e.g., “I know where to go for help”), (2) strength (refers to an individual’s recovery after setbacks and past experiences and the ability to become strong, e.g., “Coping with stress makes me feel empowered”), (3) optimism (reflects the individual’s tendency to see the positive side of things and trust their own personal and social resources, e.g., “I can cope no matter what happens”). The questionnaire uses a five-point Likert scale ranging from 1 (completely disagree) to 5 (completely agree). Responses across the 25 items were averaged to obtain the score, with higher scores indicating a higher capacity for resilience (Yu and Zhang, 2007). Studies have proved that it has good reliability and validity in the context of Chinese culture (Yang et al., 2019). In this study, the second-order CFA model generated a very good fit, with χ^2^/*df* = 4.299, *p* < 0.001, SRMR = 0.057, RMSEA = 0.052, GFI = 0.914, and TLI = 0.948, and both the absolute and value-added adaptation indexes were in the acceptable range. The Cronbach’s alpha coefficient for the Connor-Davidson Resilience Scale was 0.912.

#### Control Variable

Previous studies have shown that gender and age have a significant effect on learning burnout (Pang et al., 2010; Worly et al., 2018). To exclude the possible effect of these variables on the relationship between dependent and target variables, this study uses them as control variables to test the study hypothesis more precisely.

### Statistical Analysis

Organize and analyze data using SPSS 23.0 and SPSS macro program PROCESS compiled by Hayes (2013). The subjective well-being mediating effect is first analyzed using model 4 of PROCESS, and then whether the direct effect and the mediating effect are moderated is tested using model 59 (Bolin, 2014). A bias-corrected percentile Bootstrap (resampled 5,000 times) was used to test the moderated mediation model.

The construct validity of the scale and Common Method Deviation Test were tested using Mplus 8.0. For the goodness of fit, two classes of indexes (i.e., statistical indicators reflecting the degree of fit between the hypothesized conceptual model and the empirical data) were adopted: absolute fit and relative fit measures. The formerly included χ^2^ and normed-χ^2^ (NC), where a non-statistically significant χ^2^ value and NC values of under 3.0 indicate a good fit (Schafer and Graham, 2002; Hair et al., 2011). The latter comprised comparative fit index (CFI), goodness of fit index (GFI), normed fit index (NFI), Tucker-Lewis coefficient (TLI), and the root mean square error of approximation (RMSEA). Thresholds for good model fit were: CFI > 0.90, GFI > 0.90, NFI > 0.90, TLI > 0.90, RMSEA < 0.08 (Marsh and Hau, 1996; Schermelleh-Engel et al., 2003).

## Results

### Common Method Deviation Test

The common method deviation was controlled by anonymous, different measurement forms and scoring methods, and two methods were used to test the common method deviation. (1) Harman single-factor method is used for common method deviation testing. After principal component analysis, 16 eigenvalues greater than 1 were extracted. The first factor explaining the variance is 20.483%, which was lower than the 40% required by the critical standard (Jia et al., 2017), indicating that the questionnaire used in this study did not have a significant common method deviation problem. (2) A confirmatory factor analysis of the single-factor model of all measurement items shows that the fitting indicators are not ideal, χ^2^*/df* = 6.937, CFI = 0.325, NFI = 0.297, TLI = 0.281, RMSEA = 0.104. Therefore, there is no serious common method deviation in this study (Xiong et al., 2013).

### Descriptive Statistics and Correlation Analysis

The results of the gender difference test showed that boys’ learning burnout score (*M* = 2.829, SD = 0.478) was significantly higher than that of girls (*M* = 2.742, SD = 0.422), *t*(548) = 2.203, *p* < 0.05, *d* = 0.087. The gender difference in subjective well-being is not significant (*M*_*male*_ = 5.523, SD*_*male*_* = 2.066; *M*_*female*_ = 5.814, SD*_*female*_* = 2.269), *t*(548) = −1.497, *p* > 0.05; gender difference in resilience is not significant (*M*_*male*_ = 2.572, SD*_*male*_* = 0.474; *M*_*female*_ = 2.542, SD*_*female*_* = 0.425), *t*(548) = 0.760, *p* > 0.05. The results of the correlation analysis (see Table 2) show that the relationship between the main research variables (gender and age as the control variables) is generally in line with the hypothesis of this research, and the correlation coefficients range between −0.411 and 0.501 (*p* < 0.001). Among them, FSES is significantly positively correlated with subjective well-being and resilience, and significantly negatively correlated with learning burnout; subjective well-being is significantly negatively correlated with learning burnout, and resilience is significantly negatively correlated with subjective well-being and learning burnout.

**TABLE 2:** Descriptive statistics and correlations for primary study variables (*r*, *n* = 550).

Variables	*M*	SD	1	2	3	4	5	6
1. Gender	0.640	0.481	1					
2. Age	19.927	1.559	−0.176**	1				
3. FSES	0.014	3.624	−0.089*	0.009	1			
4. Subjective well-being	2.774	0.445	0.064	0.033	0.193**			
5. Learning burnout	5.708	2.199	−0.094*	−0.120**	−0.187**	−0.383**		
6. Resilience	2.553	0.444	−0.032	0.105*	0.185**	0.501**	−0.411**	1

Gender is a dummy variable, male is 1 and female is 0; *p < 0.05, **p < 0.01.

### Mediating Effect Test

The mediating effect of subjective well-being between FSES and learning burnout was tested under the condition of controlling gender and age using model 4 of PROCESS. The results are shown in Table 3, with FSES significantly negatively predicting learning burnout (*β* = −0.194, *p* < 0.001) and positively predicting subjective well-being (*β* = 0.200, *p* < 0.001). When FSES and subjective well-being both predict learning burnout, subjective well-being has a significant negative predictive effect on learning burnout (*β* = −0.340, *p* < 0.001), and the negative predictive effect of FSES on learning burnout is still significant (*β* = −0.126, *p* < 0.01). The results of the mediating effect test show that subjective well-being plays a mediating role in FSES’ prediction of learning burnout, with an effect size value of −0.068 and a 95% Bootstrap confidence interval of [−0.107, −0.035] (excluding 0), the mediating effect accounts for 35.052% of the total effect. H1 was validated.

**TABLE 3:** Mediating model test of subjective well-being.

Regression equation		Overall fit index			Significance of regression coefficient			
		
Outcome variable	Predictor variable	*R*	*R* ^2^	*F*	*β*	CI lower	CI upper	*t*
Learning burnout		0.259	0.067	13.049***				
	Gender				−0.280	−0.449	−0.110	−3.235**
	Age				−0.009	−0.142	−0.038	−3.379***
	FSES				−0.194	−0.273	−0.114	−4.778***
Subjective well-being		0.215	0.046	8.782***				
	Gender				0.188	0.013	0.363	2.113*
	Age				0.031	−0.023	0.084	1.119
	FSES				0.200	0.118	0.282	4.479***
Learning burnout		0.426	0.182	30.251***				
	Gender				−0.215	−0.375	−0.056	−2.650**
	Age				−0.079	−0.128	−0.030	−3.182**
	Subjective well-being				−0.340	−0.417	−0.264	−8.744***
	FSES				−0.126	−0.202	−0.050	−3.242**

The standard score was used for each variable in the model. The lower limit of CI and the upper limit of CI refers to the lower and upper limits of the 95% confidence interval (Confidence Interval), respectively. *p < 0.05, **p < 0.01, ***p < 0.001.

### Moderated Mediation Model Test

**FIGURE 2:**
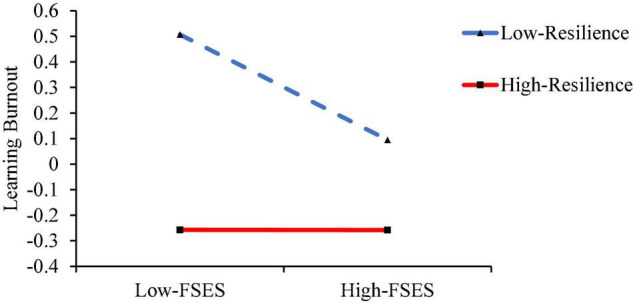
Moderating role of resilience in the relationship between FSES and learning burnout.

**FIGURE 3:**
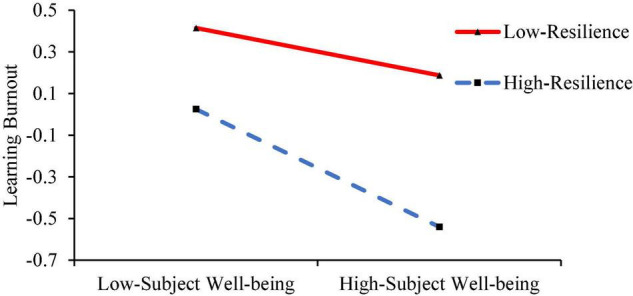
Moderating role of resilience in the relationship between subjective well-being and learning burnout.

Under the condition of controlling gender and age, the moderating effect of resilience is tested using model 59. The results are shown in Table 4. The product of FSES and resilience significantly positively predicts learning burnout (*β* = 0.104, *p* < 0.01) and predicts subjective well-being insignificantly (*β* = 0.040, *p* > 0.05), and the product of subjective well-being and resilience significantly negatively predicts learning burnout (*β* = −0.086, *p* < 0.05). This indicates that the predictive effects of FSES and subjective well-being on learning burnout are both regulated by resilience. H2 was validated. The simple slope test is further used to analyze the moderating effect of resilience in FSES and learning burnout, as well as subjective well-being and learning burnout. The mental resilience score is higher than *M* + SD as the high group, and the lower than *M* − SD as the low group, and the group regression is performed. The results are shown in Figures 2, 3. As the level of resilience increases, the negative direct effect of FSES on learning burnout gradually weakens (from *β* = −0.204, *p* < 0.001 to *β* = −0.001, *p* = 0.993), which is also shown in Table 5. What is more, the negative predictive effect of subjective well-being on learning burnout gradually increased from insignificant (*β* = −0.113, *p* = 0.063) to significant (*β* = −0.281, *p* > 0.001). Based on that, the present study shows the mediating effect of subjective well-being in Table 6 when the resilience is at three levels of the *M* − SD, *M*, and *M* + SD.

**TABLE 4:** Moderated mediating model test.

Regression equation		Overall fit index			Significance of regression coefficient			
		
Outcome variable	Predictor variable	*R* ^2^	*R* ^2^	*F*	*β*	CI lower	CI upper	*t*
Subjective well-being		0.521	0.271	40.456***				
	Gender				0.188	0.035	0.341	2.411*
	Age				−0.003	−0.051	0.044	−0.132
	FSES				0.107	0.034	0.181	2.867**
	Resilience				0.488	0.411	0.565	12.417***
	FSES × resilience				0.040	−0.026	0.106	1.202
Learning burnout		0.501	0.251	25.912***				
	Gender				−0.219	−0.373	−0.065	−2.794**
	Age				−0.068	−0.115	−0.020	−2.805**
	Subjective well-being				−0.102	−0.176	−0.028	−2.719**
	FSES				−0.197	−0.282	−0.112	−4.560***
	Resilience				−0.286	−0.373	−0.198	−6.429***
	FSES × resilience				0.104	0.030	0.178	2.772**
	Subjective well-being × resilience				−0.086	−0.159	−0.013	−2.300*

The standard score was used for each variable in the model. The lower limit of CI and the upper limit of CI refers to the lower and upper limits of the 95% confidence interval (Confidence Interval), respectively. *p < 0.05, **p < 0.01, ***p < 0.001.

**TABLE 5:** Direct effects of resilience’s different levels.

Resilience	Direct effect size	Boot standard error	CI lower	CI upper	*t*
−1 (*M* − SD)	−0.204	0.054	−0.310	0.099	−3.802***
0	−0.102	0.038	−0.176	−0.028	−2.719**
1 (*M* + SD)	−0.001	0.051	−0.102	0.101	−0.009

The standard score was used for each variable in the model. The lower limit of CI and the upper limit of CI refers to the lower and upper limits of the 95% confidence interval (Confidence Interval), respectively. **p < 0.01, ***p < 0.001.

**TABLE 6:** Mediating effects of resilience’s different levels.

Resilience	Indirect effect size	Boot standard error	CI lower	CI upper
−1 (*M* − SD)	−0.008	0.008	−0.026	0.001
0	−0.021	0.009	−0.039	−0.005
1 (*M* + SD)	−0.041	0.017	−0.077	−0.012

*The standard score was used for each variable in the model. The lower limit of CI and the upper limit of CI refers to the lower and upper limits of the 95% confidence interval (Confidence Interval), respectively.*

To sum up, subjective well-being plays a mediating role between FSES and learning burnout, and the direct effect of FSES on learning burnout and the mediating effect of subjective well-being are moderated by resilience in the second half. Specifically, with the increase in resilience level, the direct effect of FSES on learning burnout is decreasing, and the mediating effect of subjective well-being is increasing.

## Discussion

Based on previous research and the family stress model and psychological resilience framework theory, the present study constructs a moderated mediation model based on subjective well-being as a mediating variable and resilience as a moderating variable, which not only clarifies the problem of FSES “how to affect” college students’ learning burnout (the mediating role of subjective well-being) but also responses what conditions for FSES did the more significant effects of learning burnout (the moderating effect of resilience). The results of the study have some theoretical and practical significance for deepening the relationship between FSES and individual psychological and behavioral adaptation, guiding college students with poor family conditions to cultivate subjective well-being and alleviating learning burnout.

Consistent with previous studies, the results of the study show that the FSES is significantly negative in predicting college students’ learning burnout (Luo et al., 2016; Yun et al., 2016). Families with lower socioeconomic status provide less available capital for their children’s development, and their children lack opportunities to enjoy high-quality educational resources. The pressure from the family’s social economy also prevents their children from devoting themselves to learning, which may affect their learning adaptation (Wei, 2015), and may even lead to desertion, runaway, and other rebellious behavior. As a result, lower socioeconomic status affects family function and individual adaptation, and children from low-economic families are more likely to experience learning burnout (Masarik and Conger, 2017). What is more, learning burnout affects college students’ academic achievement and learning efficiency, and even leads to individuals experiencing stress events such as failing courses, repeating grades, or dropping out of school, so that individuals experience strong frustration, depression, indifference, anxiety, depression, low sense of self-worth, and other negative psychological state, seriously affecting their mental health, subjective well-being, and life satisfaction (Li and Tan, 2007; Shan et al., 2010; Qian et al., 2015).

The study found that FSES affected college students’ learning burnout by partially mediating role of subjective well-being, and generally supported the family stress model that high FSES college students were more able to be satisfied in material and life, experience higher subjective well-being, and therefore more active in learning, self-efficacy and achievement (Neubauer et al., 2018; Buzzai et al., 2020), which supports H1. Subjective well-being reflects the individual’s life satisfaction and emotional level, and people with high subjective well-being think that life is full of hope and their emotions are more positive (Conger et al., 2010). Therefore, although low FSES has a negative effect on college students’ burnout, they can reduce the level of burnout by improving their subjective well-being.

Based on the psychological resilience framework theory, the present research investigates the moderating role of resilience in the relationship between FSES, subjective well-being, and college students’ learning burnout. The results found that resilience can not only moderate the relationship between FSES and learning burnout but also moderate the mediating model of “FSES—subjective well-being—learning burnout,” which supports H2. Specifically, compared with individuals with high resilience, the direct predictive effect of FSES on learning burnout is more significant for individuals with low resilience. This result not only shows that there are individual differences in the process of resilience causing learning burnout (the mediating role of subjective well-being) but also shows that resilience is a protective factor against other factors to cause individuals to emerge with social maladjustment, which is consistent with the results of previous studies (Stoiber and Good, 1998; Xiaoyi et al., 2004). According to the psychological resilience framework theory, resilience can help individuals change their dangerous environmental factors or make selective awareness of the environment, intentionally or unintentionally, and use this interaction process between the individual and the environment to help high-risk teenagers to control the high-risk environment. Transform into a relatively protective environment, which includes selective perception, cognitive reframing, planning and dreaming, identification and interaction with pro-social people, and positive changes to the environment and proactive response, etc. (Kumpfer, 1999). Individuals with low resilience lack the above-mentioned abilities, and thus it is difficult to get rid of the negative influence of risk environment factors on learning burnout during the growth process. Their learning burnout level increases with the decrease of FSES, while resilience buffers the risk situation. For the impact of problem behaviors, the level of learning burnout remains relatively stable and low.

In addition, the study found that subjective well-being had a positive effect on the learning burnout of high resilience college students compared to low resilience individuals. The results show that resilience, as a cognitive factor, can regulate the influence of emotional factors (subjective well-being) on individual problem behavior, that is, the negative predictive effect of subjective well-being on learning burnout requires a higher resilience of individuals. The psychological resilience framework theory suggests that subjective well-being and resilience, as the process of interaction between internal psychological elasticity factors and negative environmental factors in the body, form a process of psychological resilience and determine the behavioral outcome of individuals (Kumpfer, 1999). College students with low FSES will experience more learning burnout problems, which means that under the negative effect of FSES and the positive influence of protective factors of resilience at the same time, subjective well-being from the internal emotional factors of college students through the internal intermediary process to enable individuals to obtain the final development results, and this result is a bad adaptive result. The lower resilience of individuals can not completely alleviate the negative effects of low FSES, nor can they give full play to the positive effects of emotional factors on learning burnout, which ultimately leads to lower subjective well-being and higher learning burnout (Seligman et al., 2009).

The present study had important theoretical and practical implications for developing interventions for learning burnout. On the theoretical aspect, this result enriches the complex mechanism of interaction between emotional and cognitive factors and individual development results in the existing psychological resilience framework theory, and on the other hand, it provides a theoretical basis and a new perspective for the intervention of positive emotions to promote individual learning adaptation in the practice of real mental health education. The main findings of this study are important for the development of interventions for learning burnout. FSES can indirectly play a role in college students’ learning burnout through the mediating path of subjective well-being, while resilience can regulate the predictive effect of FSES and subjective well-being on learning burnout. This also suggests that learning burnout intervention for college students from different family backgrounds needs to pay attention to the combination of individual cognitive factors and emotional factors for educators and parents through the cultivation of good resilience to increase the positive effect of subjective well-being on learning burnout.

## Limitations

The following limitations exist in this study. First of all, this study uses the cross-sectional study design, which cannot make a causal inference. This moderated mediation model proposed by the present study can be further tested by longitudinal research in the future. Secondly, this study measures resilience through The Connor-Davidson Resilience Scale, preferring to regard resilience as a trait and neglecting to pay attention to the process of mental resilience. Future research can manipulate different ways of psychological resilience by the experimental method, thus inducing different forms of psychological resilience process of individuals, to examine the moderating effect of resilience on the relationship between FSES, subjective well-being, and learning burnout. Thirdly, the variables in this study are self-reporting. Future studies can measure the main variables of this study using a variety of methods (e.g., self-reporting, parental reporting, and teacher evaluation). In addition, the sample in this study is university students from Kunming, which may affect the popularity of the results. Future researchers can select college students from different regions, majors, and ages to conduct surveys to further test the main results of this study.

## Conclusion

(1) After controlling for participants’ gender and age, FSES negatively, and significantly predicted learning burnout;
(2) Subjective well-being partially mediated the relationship between FSES and learning burnout;
(3) The direct effect of FSES on learning burnout and the mediation effect of subjective well-being was moderated by resilience. The level of learning burnout of individuals with low resilience increased significantly with the decrease of FSES, and the level of learning burnout of individuals with high resilience decreased significantly with the increase in subjective well-being.

## Data Availability Statement

The datasets presented in this study can be found in online repositories. The names of the repository/repositories and accession number(s) can be found in the article/Supplementary Material.

## Ethics Statement

The questionnaire and methodology for this study was approved by the Human Research Ethics Committee of the Kunming Medical University (Ethics approval number: 2021kmykdx6f65). Written informed consent to participate in this study was provided by the participants’ legal guardian/next of kin. Written informed consent was obtained from the individual(s), and minor(s)’ legal guardian/next of kin, for the publication of any potentially identifiable images or data included in this article.

## Author Contributions

WW, YZ, and XL: research idea and study design. ZCG, XC, XW, XZ, and FT: data collection. WW, YL, XM, and LY: data analysis and manuscript writing. ZYG, SL, YC, and QL: supervision, project administration, and funding acquisition. All authors contributed to the article and approved the submitted version.

## Conflict of Interest

The authors declare that the research was conducted in the absence of any commercial or financial relationships that could be construed as a potential conflict of interest.

## Publisher’s Note

All claims expressed in this article are solely those of the authors and do not necessarily represent those of their affiliated organizations, or those of the publisher, the editors and the reviewers. Any product that may be evaluated in this article, or claim that may be made by its manufacturer, is not guaranteed or endorsed by the publisher.
